# The Immunopathology of COVID-19 and the Cannabis Paradigm

**DOI:** 10.3389/fimmu.2021.631233

**Published:** 2021-02-12

**Authors:** Nicole Paland, Antonina Pechkovsky, Miran Aswad, Haya Hamza, Tania Popov, Eduardo Shahar, Igal Louria-Hayon

**Affiliations:** ^1^Medical Cannabis Research and Innovation Center, Rambam Health Care Campus, Haifa, Israel; ^2^Clinical Immunology Unit, Rambam Health Care Campus, Haifa, Israel; ^3^Clinical Research Institute at Rambam (CRIR), Rambam Health Care Campus, Haifa, Israel; ^4^Department of Hematology, Rambam Health Care Campus, Haifa, Israel

**Keywords:** SARS-CoV2, COVID-19, cytokine release syndrome, cytokine storm, inflammation, cannabis, cannabinoids, cannabinoid receptors

## Abstract

Coronavirus disease-19 caused by the novel RNA betacoronavirus SARS-CoV2 has first emerged in Wuhan, China in December 2019, and since then developed into a worldwide pandemic with >99 million people afflicted and >2.1 million fatal outcomes as of 24th January 2021. SARS-CoV2 targets the lower respiratory tract system leading to pneumonia with fever, cough, and dyspnea. Most patients develop only mild symptoms. However, a certain percentage develop severe symptoms with dyspnea, hypoxia, and lung involvement which can further progress to a critical stage where respiratory support due to respiratory failure is required. Most of the COVID-19 symptoms are related to hyperinflammation as seen in cytokine release syndrome and it is believed that fatalities are due to a COVID-19 related cytokine storm. Treatments with anti-inflammatory or anti-viral drugs are still in clinical trials or could not reduce mortality. This makes it necessary to develop novel anti-inflammatory therapies. Recently, the therapeutic potential of phytocannabinoids, the unique active compounds of the cannabis plant, has been discovered in the area of immunology. Phytocannabinoids are a group of terpenophenolic compounds which biological functions are conveyed by their interactions with the endocannabinoid system in humans. Here, we explore the anti-inflammatory function of cannabinoids in relation to inflammatory events that happen during severe COVID-19 disease, and how cannabinoids might help to prevent the progression from mild to severe disease.

## Introduction

**Figure 1:**
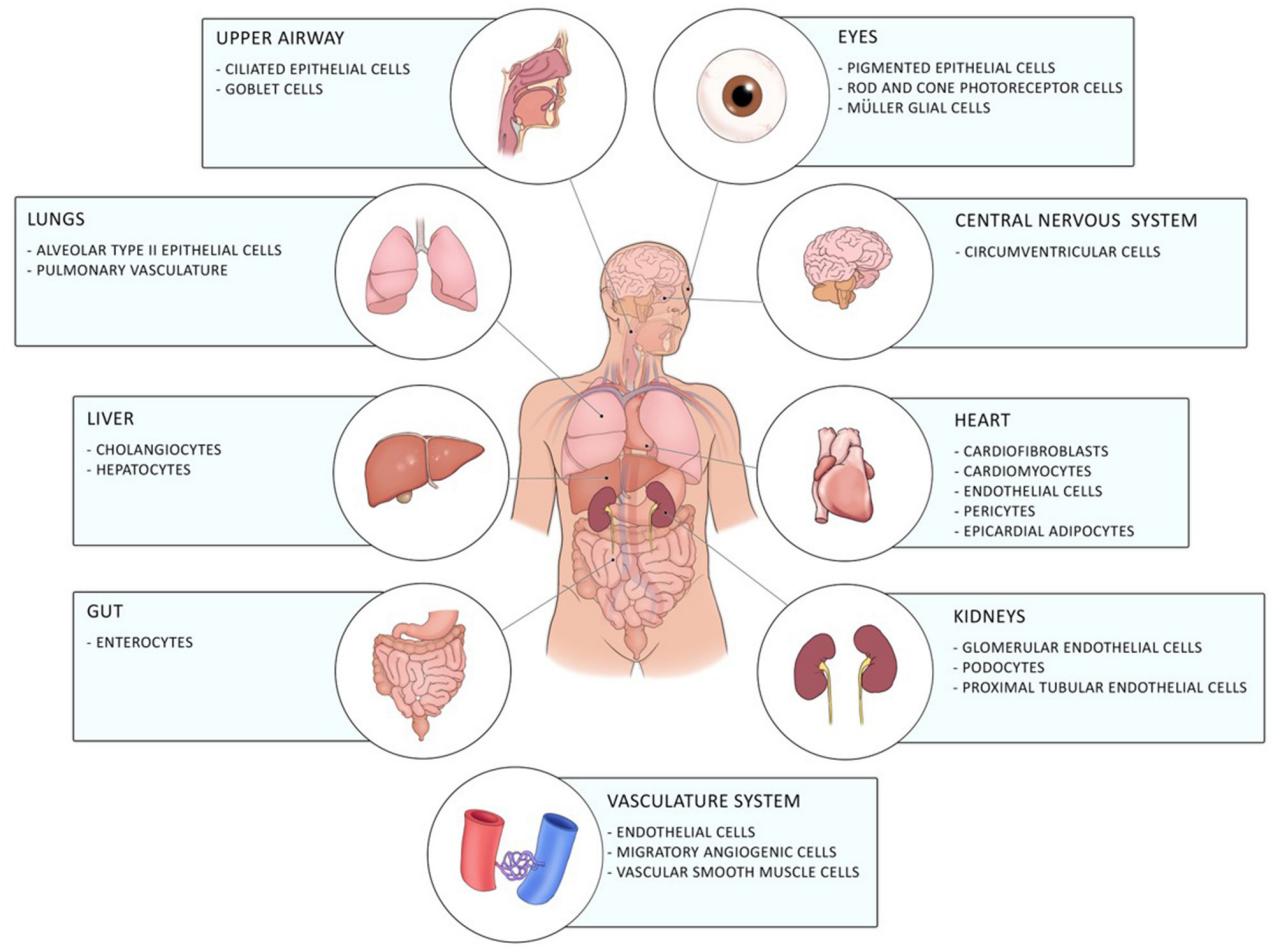
Distribution of ACE2 in the human body. ACE2 is expressed in different cells of the eye, the upper airway, the lung, the liver, the gut, the central nervous system, the heart, the vasculature system, and of the kidneys.

Coronavirus disease 19 (COVID-19) caused by the novel severe acute respiratory syndrome-coronavirus 2 (SARS-CoV2) firstly emerged in December 2019 in Wuhan in China and has, since then, evolved into a global pandemic (1). It is a novel enveloped RNA betacoronavirus, which binds with its spike surface protein (S-protein) to angiotensin-converting enzyme 2 (ACE2) on the cellular host's surface. Entry of the virus to the host cell by endocytosis requires cleavage of the S-protein by the host cell transmembrane protease serine-2 (TMPRSS-2) (2). ACE2 is expressed in a diverse array of cells including cells of the upper respiratory, central nervous and vasculature system as well as of the eye, lung, liver, heart, kidney, and intestine contributing to the diverse clinical pulmonary and extra-pulmonary manifestations of COVID-19 including gastrointestinal involvement of COVID-19 [(3–5); Figure 1].

SARS-CoV2 similarly to other coronavirus outbreaks SARS-CoV1 and Middle East Respiratory Syndrome (MERS) targets the lower respiratory tract system leading to pneumonia with fever, cough, and dyspnea (6). Most patients (80%) show only mild disease (either no or mild pneumonia), a smaller proportion (14%) develops severe symptoms with >50% pulmonary manifestations as observed on imaging tests including dyspnea and hypoxia. A small proportion (5%) develop a critical disease with respiratory failure, multi-organ failure or systemic shock. About 10–30% of hospitalized patients get into a critical stage where they require intensive care for respiratory support. Approximately 1% of all patients have a fatal outcome (7, 8). Patients who developed acute respiratory distress syndrome (ARDS) and required mechanical ventilation had a reported mortality rate of 88.1% in the New York City area in March 2020 (9). Interestingly, not only elderly, but also young patients with only mild comorbidities like hypertension, diabetes mellitus and obesity developed respiratory failure (10).

Epidemiological data show that differences in susceptibility and severity of COVID-19 widely depend on biological and socio-economic factors. A lower incidence of severe disease was observed in females with caucasian heritage (11, 12). This might be due to the fact that females are less susceptible to viral infections due to a higher macrophage and neutrophil activity and an increased antibody production and lower cytokine production (12). On the other hand seem ethnic minorities more susceptible to contract a SARS-CoV2 infection (11, 12). Ethnic minorities usually have less access to a functioning health care system, higher levels of medical comorbidities, and their lower socioeconomic status contributes to a weak cell-mediated immunity (11, 12).

**Figure 2:**
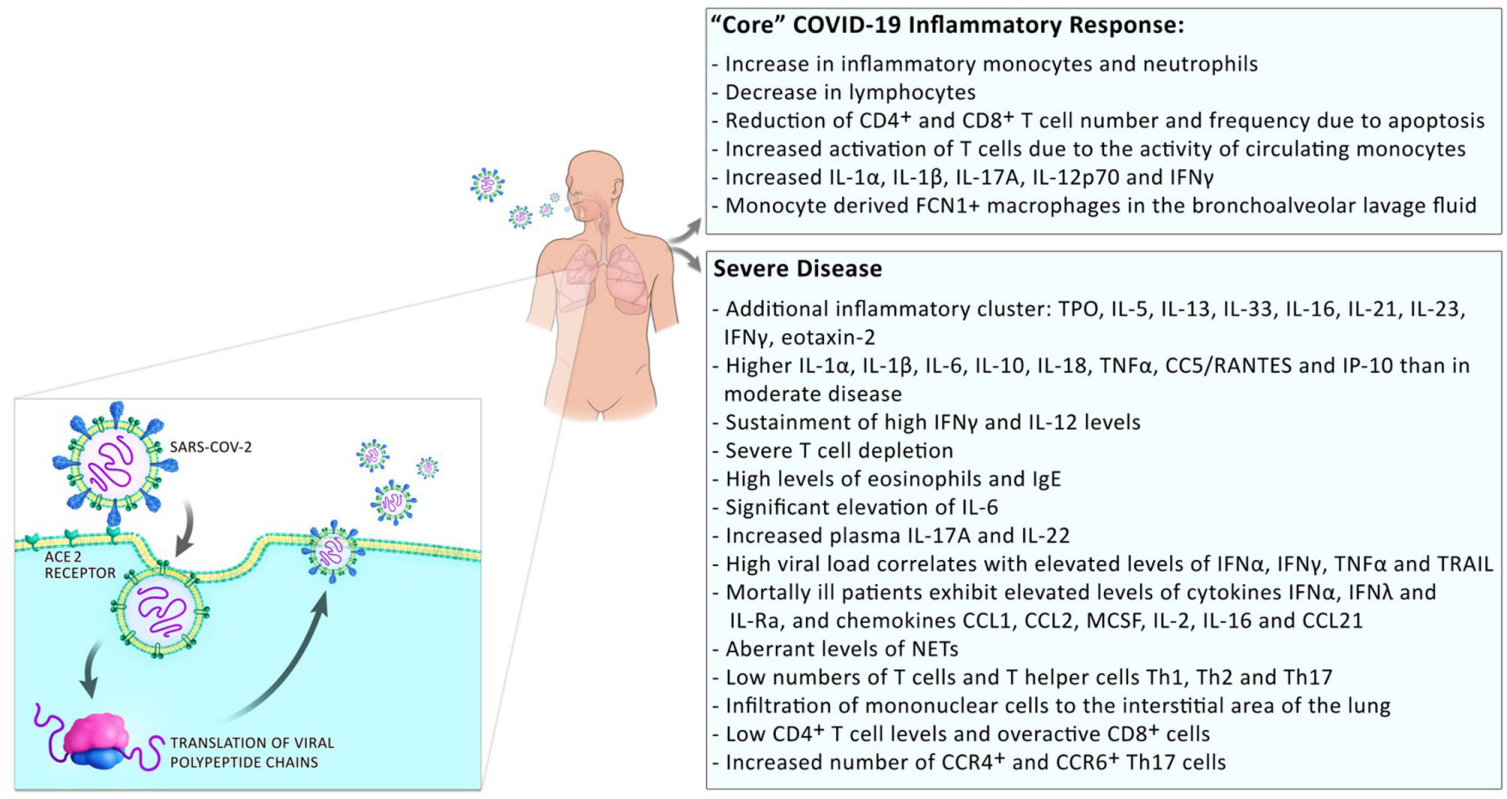
Inflammatory responses in COVID-19 patients. Inflammatory responses to SARS-CoV2 comprise a “core” inflammatory response, which all COVID-19 patients experience. In patients with mild disease, the inflammatory response resolves on the way to convalescence. The appearance of additional inflammatory clusters is observed during progression to severe disease. This includes the release of a higher number of systemic pro-inflammatory cytokines, low numbers but over-reactive T cells, and infiltration of monocytes/macrophages to the sites of infection.

Development of a severe disease progresses in two steps; mild symptoms at the start are followed by respiratory worsening after ~10 days after onset of initial symptoms. This deterioration is accompanied with clinical presentations of ground-glass lung opacities on chest imaging, lymphocytopenia, high D-dimer, and high prothrombin (1). In patients with moderate disease, a progressive reduction in inflammatory responses happens in convalescence while in patients with severe disease, these levels remain high and an additional cluster of inflammation appears (13) possibly due to a defective type I interferon response [(14); Figure 2]. Those systemic hyperinflammatory patterns in COVID-19 patients are similar to those in cytokine release syndrome (CRS) and the occurrence of CRS in severe disease was suggested (15).

Although certain patterns for the susceptibility to develop severe COVID-19 are recognizable (13, 16), the inflammatory response and the immune dynamics of the infected patient are still not fully understood. This makes the exact course of a SARS-CoV2 infection and the correlation to a certain clinical manifestation highly unpredictable. Effective and specific anti-viral drugs against SARS-CoV2 are not yet available. The usage of available repurposed anti-inflammatory medicines (17) and antibody-based immunotherapeutics targeting viral clearance (18, 19) is still experimental and applied only for the treatment of severely and critically ill patients (20–22). Safe and efficient treatment options that have the potential to halt disease progression at an early stage are needed. Cannabis and cannabinoids with their well-known anti-inflammatory properties may hold this potential.

Cannabis comprises various strains termed *Cannabis sativa, Cannabis ruderalis*, and *Cannabis indica*. It is not sure if they are three different species or whether ruderalis and indica are subspecies of *C. sativa*. During the history of the mankind, the cannabis plant was grown for varied uses as for production of fabric, for food, for recreational purposes and for medicinal use. Medically useful substances are produced in the trichomes that sit on the leaves and buds of the plant (23).

The cannabis plant contains more than 550 different components, of which are about 150 belongs to C21 or C22 terpenophenolic phitochemicals, which are predominantly expressed in the cannabis plant thus termed cannabinoids. The other 400 components are terpenes and phenolic compounds (24). The cannabinoids components contains both psychoactive [as 9-tetrahydrocannabinolic acid (THCA)] and non-psychoactive [as cannabidiolic acid (CBDA)] substances (24). The biological properties of the cannabinoids rely on their interaction with the endocannabinoid system includes G-proteins coupled receptors and Transient receptor potential chanels (TRP) (25).

Most interestingly, the anti-inflammatory properties especially of the non-psychoactive cannabidiol were recently explored as anti-viral agents. These effects were shown for the treatment of HIV (26), viral hepatitis (27), or influenza (28, 29) as well as orthopoxvirus, borna disease virus or vaccinia virus (30).

Here, we will explore the anti-inflammatory qualities of phytocannabinoids and discuss the possibility of applying cannabinoids as a treatment option for COVID-19 patients. We will explore the recent literature and emphasize the anti-inflammatory properties in relation to the events occurring during cytokine release syndrome (CRS) in mild or severe COVID-19 disease.

## Cytokine Release Syndrome in Covid-19 Patients

Each infection with SARS-CoV2 is closely related to excessive inflammatory events with monocytes/macrophages and T cells playing a special role (31). Diffuse alveolar damage, pulmonary thrombi and vasculitis occurring predominantly in monocytes and myeloid tissue and an excess of plasma cells in lymph nodes, spleen, and lung was observed (15, 32). This leads to a severe increase in white blood cells with concomitant decrease in CD4^+^ and CD8^+^ lymphocytes resulting in an impaired neutrophil to lymphocyte ratio (33). Inflammatory cells infiltrate the sites of infection at an early stage of the infection with SARS-CoV2 and cause a stormy release of pro-inflammatory cytokines like IL-6, IL-17A, TNFα, IFNγ, IL-1α/β, and chemokines like CC-chemokine ligand 2 (CCL2) as well as CXC-chemokine ligand 10 (CXCL10) (13). The release of IL-6 by circulating monocytes into the bloodstream leads to an increased activation of T cells with a concomitant reduced total number of T cells. Monocyte-derived FNC1+ macrophages were found in bronchoalveolar lavage fluid (BALF) (13, 34) probably contribute to the hyperinflammation phenomenon (35). CRS can develop at every stage of infection beginning with the entry of the virus into the host causing innate and adaptive immune responses.

### Induction of CRS During SARS-CoV2-ACE2 Interaction

Binding of the virus to ACE2 leads to the internalization of ACE2 and activation of angiotensin II resulting in the activation of nuclear factor kappa B (NF-κB). Subsequently, cytokines IL-6, TNFα, IL-1β, and IL-10 will be produced which might lead to local lung dysfunction including a rise in blood pressure, which contributes to lung injury and deterioration of pulmonary function as occurs in ARDS (36, 37).

### Induction of CRS by Innate Immune Cells

ACE2 was found on CD169^+^ macrophages in lymph nodes and spleen of COVID-19 patients and severe lymphocyte apoptosis was observed probably induced by viral antigens through Fas upregulation (38). CD169^+^ cells control viral replication via type I interferon, and expose viral antigens to recognition by adaptive immune cells (39). Infection of CD169^+^ macrophages enables the translocation of the virus to the spleen and lymph nodes, which contributes to the body-wide distribution of the virus resulting in accumulation of pro-inflammatory monocytes and macrophages at the sites of infection and at sites adjacent to the infection (40).

Monocyte derived *FCN1*+ macrophages that produce pro-inflammatory cytokines and chemokines were found in the BALF of patients with ARDS (34). This might contribute to the induction of T cell apoptosis, which might lead to pneumonia and disease progression to ARDS (41). Moreover, production of IL-6 in the spleen and lymph nodes, and of IL-6, TNFα, IL-10, and PD-1 by alveolar macrophages induces lymphocyte necrosis, further contributing to the development of lymphocytopenia and cytokine storm in the lung (38).

Monocytes and mononuclear cells in the peripheral blood and BALF are activated and secrete IL-6, IL-10, and TNFα, and chemoattractors of macrophages' IFN-induced protein 10 (IP-10), and MCP-1 (42). In peripheral blood mononuclear cells (PBMC) and BALF, high levels of neutrophil-attracting chemokines CXCL2 and CXCL8 attract neutrophils to the site of inflammation (43). Neutrophils secrete extracellular webs of DNA and histone to infectious particles, termed NETs, which are found aberrantly in patients with ARDS (44). They are believed to contribute to venous and arterial thrombosis in critical disease, multi-organ, and respiratory failure as well as coagulopathy due to their impact on the regulation of cytokine release (42).

### Induction of CRS by Adaptive Immune Cells

Lymphocytopenia is a well-observed symptom in patients with severe COVID-19. It comprises a highly reduced number of circulating B and T cells combined with an increase in neutrophils and hyperactivation of monocytes and macrophages.

Patients with severe disease symptoms have low T cells and T helper cells, Th1, Th2, and Th17 numbers (45), either due to T cell apoptosis by high amounts of cytokines secreted by CD4^+^ T cells (46, 47) or due to the redistribution to other tissues resulting in the infiltration of mononuclear cells into the interstitial area of the lung contributing to the development of interstitial pneumonitis (48).

In patients with ARDS, CD4^+^, and CD8^+^ T cells were found in the peripheral blood 14 days after disease onset. While CD8^+^ T cells secrete primarily IFNγ, CD4^+^ T cells secrete cytokines related to Th1 (IFNγ, TNFα, IL-2) and Th2 (IL-5, IL-9, IL-10) at normal levels (49) albeit at reduced levels in severe COVID-19 (50). Patients with ARDS had low but over-activated CD8^+^ cells (51) leading to T cell exhaustion rendering the T cell response ineffective (50). In addition, activated T cells, including Th1 and Th17 helper cells, further stimulate the activation of monocytes enabling secretion of IL-1β, IL-6, and colony stimulating factor (CSF1 and CSF2) to contribute to the worsening of the cytokine storm resulting in organ failure (46).

Another important subset of T cells comprises regulatory T cells (Tregs). They are responsible for regulating the immune response to prevent hyperinflammation. To do so, they expand rapidly in antiviral immune responses (52). Inconsistent results have so far been obtained about the levels of Tregs in patients with severe COVID-19. Some observed higher levels of Tregs, while others reported reduced or unchanged levels (45).

As a result of the above, a significant elevation of a plethora of pro-inflammatory cytokine levels was reported. The most prominent elevated pro-inflammatory cytokine is IL-6 in patients with severe symptoms. This IL-6 production from CD14^+^ and CD16^+^ monocytes is driven by GM-CSF produced by Th1 cells (53) directly correlated with virus load (54). Moreover, IL-6 might influence lung-centric coagulopathy by inducing coagulation cascades (55).

Increased numbers of CCR4^+^ and CCR6^+^ Th17 cells were measured in COVID-19 patients with ARDS (56). Other cytokines IL-1, IL-17, TNFα, and GM-CSF were associated with Th17 immune responses (57). These observations might explain the occurrence of a Th17-type cytokine storm, and the onset of multiple organ damage in patients with severe COVID-19 (56). Similarly, production of IP-10, CCL5/RANTES^41^, CRP, and C-dimer were higher in patients with severe COVID-19 compared to mild COVID-19 (57). An increase of anti-inflammatory cytokines IL-10 and IL-4 hints to an elevated Th2 response, which might be involved in the development of pulmonary interstitial fibrosis (13, 58). Underlying bacterial infections might contribute to the development of CRS by exacerbating the inflammatory response (59).

## Treatment Options for Covid-19 Patients

No specific anti-viral drug for the successful treatment of COVID-19 is available. Several anti-inflammatory drugs are tested in pre-clinical and clinical trials as repurposed drugs for resolving CRS in patients with severe disease including steroids and corticosteroids like dexamethasone (60, 61), mono- and polyclonal antibodies normally used in rheumatology, i.e., the IL-6 inhibitor tocilizumab (62–65) including the “famous” monoclonal antibody cocktail REGN-COV2 (66), anti-viral drugs like remdesivir (67) or the HIV-drug combination lopinavir-ritonavir, the anti-parasitic drug hydroxychloroquine (68) as well as drugs against gastrointestinal diseases like famotidine (histamine-2 receptor antagonist), which showed antiviral properties for HIV or omeprazole (proton pump inhibitor) (69) in addition, the administration of convalescent plasma was tested (22).

Most of the drugs with the exception of remdesivir that was recently approved by the FDA, are still in clinical trials or could not reduce mortality (20–22). To prevent mortality, therapies halting disease progression at earlier stages are required. Cannabinoids with their anti-inflammatory function represent potential candidates to avoid CRS (70–72).

## Cannabis and the Endocannabinoid System

**Figure 3:**
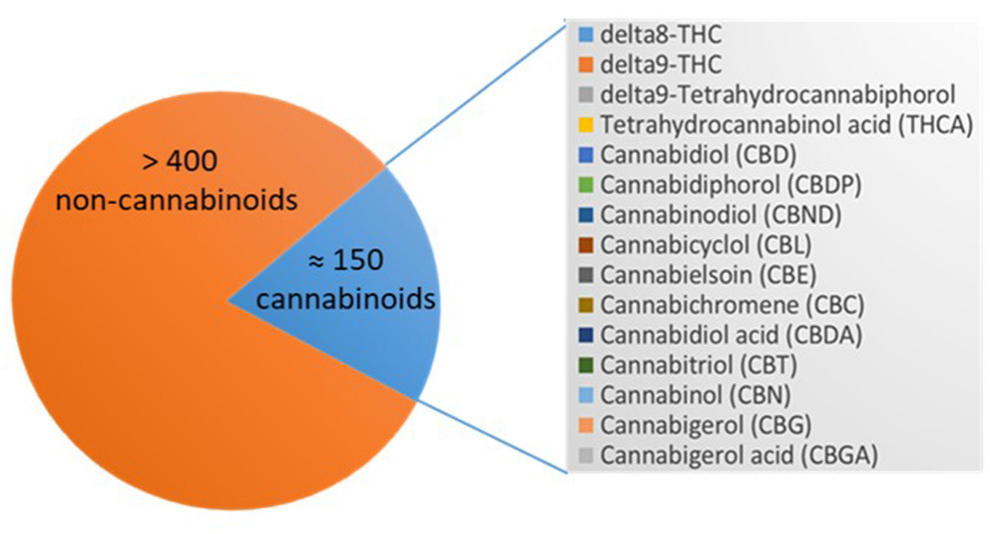
Cannabinoids in *Cannabis sativa* spp. Depicted is the number of the compounds of the cannabis plant, which are >400 non-cannabinoids and ≈150 cannabinoids that are listed including their abbreviations.

The cannabis plant comprises >550 different chemical constituents, ≈150 of these are cannabinoids and >400 non-cannabinoids. The main pharmacologically active compounds are the psychoactive tetrahydrocannabinols (THC), Δ^8^-THC and Δ^9^-THC, and other non-psychoactive cannabinoids like cannabinol (CBN), cannabidiol (CBD), or cannabigerol (CBG) to name only a few. CBN was the first cannabinoid that was isolated in 1899 (73). Non-cannabinoids are flavonoids, terpenes, and fatty acids [(23, 24); Figure 3].

### Cannabinoid Receptors and Their Ligands

Cannabinoids convey their functions via cannabinoid receptors that are anchored in the cell membrane. Cannabinoid receptors bind to endo- (eCBs) and phytocannabinoids alike comprising the endocannabinoid system. The most studied endocannabinoids are 2-arachidonoylglycerol (2-AG) (74) and N-arachidonoylethanolamide (anandamide, AEA) (75). They belong to a group of lipid mediators that are either synthesized and released from membrane phospholipids “on demand” in response to physiological or pathological stimuli by many cell types in the brain or peripheral tissue or may be stored in organelles that might serve as potential platforms for trafficking and accumulation (76).

2-AG is a monoacylglycerol that serves as an intermediate in lipid metabolism (77). It is synthesized when needed by two major pathways, a signaling and a metabolic pathway. The signaling pathway starts from phosphatidylinositol-4,5-biphosphate (PIP_2_) and the metabolic pathway starts from triglycerides that contain 2-arachidonate (78). Two second messengers are synthesized from PIP_2_: diacylglycerol (DAG) and inositol-1,4,5-triphosphate (75). Triglycerides are hydrolyzed by hormone-sensitive lipase, other lipases, and carboxylesterase to diglycerides that contain 2-arachidonylglycerol (75). Diglycerides are then further processed by two isoforms of diacylglycerol lipase α and β (DAGL-α and -β) generating 2-AG and a fatty acid (79). Degradation of 2-AG is accomplished by the hydrolysis of the ester bond into arachidonic acid and glycerol by the enzyme monoacylglycerol lipase (MAGL) or α,β-hydrolase domain-containing proteins 12 and 6 (ABHD12 and ABHD6) (80).

The biosynthesis of AEA involves two steps: (i) the formation of N-arachidonoyl-phosphatidylethanolamine from phosphatidylethanolamine catalyzed by the calcium-dependent N-acyltransferase followed by (ii) the conversion of N-arachidonoylphosphatidylethanolamine to AEA or other N-acylethanolamines through five different metabolic pathways of which the most studied pathway involves N-acyl-phosphatidylethanolamine-hydrolyzing phospholipase D (76, 81). Degradation of AEA occurs through cleavage into arachidonic acid and ethanolamine by fatty acid amide hydrolase (FAAH) or N-acylethanolamine-hydrolyzing acid amidase (82).

Both, 2-AG and AEA, can be degraded by cyclooxygenase (COX), lipoxygenase (LOX), or cytochrome P450 resulting in the formation of oxidized compounds such as hydroxyl-anandamides and hydroxyeicosatetraenloyl-glycerol or prostaglandin-ethanolamines and prostaglandin-glyceryl esters; all of them with different biological functions (76).

Two different classes of receptors are assigned as putative cannabinoid receptors, G-protein-coupled receptors (GPCRs) and transient receptor potential channels (TRP), which we present in further detail in the following two sections and in Table 1.

**Table 1:** Cannabinoids receptors.

**Receptor**	**Location in immune cells/tissue**	**Function in immunity**	**Ligands**
CB1 and CB2	- Immune tissue (spleen and thymus) and leukocyte subpopulations like natural killer cells, B-cells, and peripheral blood mononuclear cells (83, 84)	- Reduces IL-2 synthesis (85) - Suppression of T cell activation (86–89) - Inhibition of lymphocyte proliferation and IL-17 secretion (86) - Regulation of function of intestinal neutrophils (90) - Regulation of acute trafficking of neutrophils to site of inflammation (91) - Retention of immature B cells in the bone marrow (92) - Immunosuppression in B cells after AEA or Δ^9^-THC ligand binding (93)	*Endocannabinoids*: - 2-AG (94), AEA (95) *Phytocannabinoids*: - Δ^9^-THC and Δ^8^-THC as partial agonists regulating pain management (96, 97) - CBN as agonist (98) - CBD as a weak antagonist (99), as a negative allosteric modulator (100), and as an inhibitor of anandamide uptake (101) - CBG as a weak partial agonist (102, 103), and as an inhibitor of AEA uptake (101) - CBC as weak agonist (103), and as an inhibitor of AEA uptake (101) - Δ^9^-THCV as an antagonist (104)
GPR18	- Spleen (105) - CD4^+^- and CD8^+^ T cells and CD19^+^ B cells (106)	- Immune regulation in the small intestine for normal homeostasis of CD8^+^ subsets of IELs (CD8αα and CD8αβ IELs) (107)	*Endocannabinoids*: - NAGly (105) *Phytocannabinoids*: - Δ^9^-THC as an agonist (108) - CBD as antagonist (108)
GPR55	- Leukocytes	- T cell migration (109) - Hyperalgesia associated with inflammatory and neuropathic pain (110)	*Endocannabinoids*: - AEA, 2-AG, and virodhamine (111) *Phytocannabinoids*: - Δ^9^-THC as an agonist (112) and LPI inhibitor (113) - CBD as antagonist (111) - CBG as weak LPI inhibitor (113) - Δ^9^-THCV as a partial agonist and LPI inhibitor (113) - CBDV as a LPI inhibitor (113)
TRPV2	- Human and mouse B cells - Human dendritic cells and neutrophils - Mouse monocytes and macrophages	- Translocation of TRPV2 to the plasma membrane plays a role in the chemotaxis of macrophages and in phagocytosis (114) - Some analgesic and anti-proliferative properties of CBD may be mediated by TRPV2 activation (115)	*Phytocannabinoids*: - Δ^9^-THC, CBD, CBG, Δ^9^-THCV, and CBDV as agonists (101, 116)
TRPA1	- Expressed on human lung fibroblasts and epithelial cells (117)	- Agonist binding leads to release of IL-8 - Role in modulation of the release of chemokines in inflamed airways (117)	*Phytocannabinoids*:- Δ^9^-THC, CBG (118) - CBN and CBC as antagonists (101)
TRPM8	- Co-expressed with several clusters of differentiation (CD) like CD38, CD79a, CD138, and in mature B-cell neoplasms (119)	- Role in cold hypersensitivity associated with inflammatory and neuropathic pain (120)	*Phytocannabinoids*: - Δ^9^-THC, CBG (118) - CBN and CBC as antagonists (101)

*CB, cannabinoid receptor; 2-AG, 2-arachidonoylglycerol; AEA, N-arachidonoylethanolamide; NADA, N-arachidonoyl dopamine; THC, tetrahydrocannabinols; CBN, cannabinol; CBD, cannabidiol; CBG, cannabigerol; CBC, cannabichromene; THCV, tetrahydrocannabinol; GPR, G protein-coupled receptor; LPI, lysophosphatidylinositol; CBDV, cannabidivarin; NAGly, N-arachidonyl glycine; TRPV2, transient receptor potential cation channel; subfamily V; member 2; IELs, intraepithelial lymphocytes; TRPA1, transient receptor potential cation channel; subfamily A; member 1; TRPM8, transient receptor potential cation channel; subfamily M, member 8*.

#### G Protein-Coupled Receptors

G protein-coupled receptors are a family of membrane proteins. They are characterized by seven membrane-spanning α-helical domains that are separated by alternating intra- and extracellular loops. GPCRs mediate the cellular response to neurotransmitters and hormones and are mostly responsible for taste, vision, and olfaction. The most prominent GPCRs that mediate endo- and phytocannabinoid signaling believed to be involved in signal transduction of the immune system are CB1R, CB2R, GPCR18, and GPCR55 (121).

#### Transient Receptor Potential Channels

Transient receptor potential (TRP) channels are a family of ion channels. They are membrane proteins which consist of channel subunits built of six putative transmembrane-spanning segments (S1-S6) with a pore-forming loop between S5 and S6 which assemble into tetramers to form functional channels (122). TRPs are involved in the signal transduction of numerous chemical and physical stimuli and regulate many neural signaling processes and other physiological functions such as temperature sensation, smell, taste, vision, pressure, or pain perception (123). Thus, they are potentially attractive targets for the therapeutic use of phytocannabinoids in the treatment of sensory, inflammatory or dermatological pathologies (124). Most TRPs can cause channelopathies which are risk factors for many disease states (125).

TRPs that are putative cannabinoid receptors are TRPV1-4, TRPA1, and TRPM8 (126).

### Anti-inflammatory Properties of Cannabinoids and Their Potential to Downregulate COVID-19 Related CRS

First indications that cannabis has the potential to influence the disease course of COVID-19 were already published 3 years before the outbreak of the current pandemic. Researchers from Italy examined the potential of a hemp seed protein isolate that was prepared from defatted hemp seed by alkaline solubilization/acid precipitation as inhibitors for ACE-2. Four potentially bioactive peptides GVLY, IEE, LGV, and RVR were identified in the tested fraction by mono- and bidimensional NMR and LC-MS analyses. All four peptides had ACE-inhibitory activity rendering hemp seeds a potential agent to inhibit entry of SARS-CoV2 into the cells (127).

Recently, Canadian researchers have tested CBD extracts of 800 different *C. sativa* lines on 3D human models of oral, airways, and intestinal tissues and found 13 low THC/high CBD lines that modulated ACE2 and TMPRSS2 levels, which might lower the virus load (128). ACE2-reducing activity of cannabis-derived products were confirmed by a different group. They extracted a CBD, CBG, and THCV-containing fraction of a *C. sativa* strain and tested it *in vitro* in comparison to a standard phytocannabinoid agent. Both products reduced the secretion of pro-inflammatory cytokines IL-6, IL-8, CCL2, and CCL7 from the alveolar epithelial cell line A549, induced polarization of the macrophage cell line KG1 and increased the phagocytosis. CD36 and type II receptor for the Fc region of IgG (FcγRII) were upregulated. The researchers reported a certain superiority of the standard phytocannabinoids compared to the cannabis-derived fraction but cannot give recommendations for usage of cannabis in the treatment of COVID-19 (129). Another recent study simulated viral infections using the synthetic RNA Poly I:C and could show that Poly I:C-induced ARDS could be prevented by CBD (130) through the upregulation of apelin, a peptide regulating central and peripheral immunity that was severely downregulated in a murine model of ARDS (131). In the following two sections we will present the processes of regulation of the immune responses of endo- and phytocannabinoids.

#### Regulation of Immune Responses by Endocannabinoids via CB1 and CB2

The endocannabinoid system has anti-inflammatory activities in innate and adaptive immunity. It regulates migration and trafficking of different immune cells dependent on its receptors. Experiments with human bone marrow cells obtained by aspiration from healthy donors showed that the migration of human hematopoietic stem and progenitor cells was modulated by endocannabinoids. Endocannabinoids receptors CB1 and CB2 were expressed by bone marrow derived hematopoietic stem cells and CD34^+^ cells. AEA and 2-AG were detected in the microenvironment of peripheral blood and bone marrow, which were secreted by bone marrow mesenchymal stem cells. Migration of hematopoietic stem cells was stimulated by AEA and 2-AG and blocked by CB receptor antagonists rendering endocannabinoids putative candidates for the enhancement of the migration of hematopoietic stem cells (132).

Cell trafficking of mature immune and effector cells, like lymphocytes, macrophages, neutrophils, and dendritic cells can be regulated by endocannabinoids [rev. in (133)]. It was reported that exogenously added 2-AG leads to the attenuation of lymphocyte proliferation through the decrease of Th1- and Th17-associated cytokines IL-6, IL-2, and TNFα. Moreover, activated B and T cells that produce high levels of 2-AG inhibit in a feedback loop T cell activation and proliferation, making exogenously applied 2-AG a putative candidate for therapeutic usage in Th1- or TH17-dependent diseases (134). Upon antigen activation by pathogens, macrophages, and dendritic cells produce and release 2-AG, which results in the upregulation of 2-AG levels in the serum and lymph nodes of mice during vaccination CB2 dependently. In a murine immunization model, transient administration of CB2 antagonist AM630 or inverse antagonist JTE907 increased the intensity of antigen-specific immune responses by upregulation of immunomodulatory genes in secondary lymphoid tissue (135). AEA inhibited macrophage-mediated killing of the TNFα-sensitive mouse alveolar macrophage cell line L929 (136). Correa et al. presented evidence that AEA inhibited expression of pro-inflammatory cytokines like IL-12 and IL-23 in *in vitro* models of immune disorders and increased the anti-inflammatory cytokine IL-10 in activated mouse microglia (137–139). In a model of acute intestinal inflammation it was shown that the transporter p-glycoprotein helped the influx of endocannabinoids into the intestinal lumen, which inhibited the migration of neutrophils by counteracting the pro-inflammatory neutrophil chemoattractant eicosanoid hepoxilin A3 (90). Similarly, the migration-related transcriptional profile of neutrophils was enhanced in CB2^−/−^ mice. In response to Zymogen, the neutrophil, and lymphocyte antigen 6 complex was recruited to the dorsal air pouch and metalloproteinase 9 and CCL4 and CXCL10 increased (91).

#### Regulation of the Immune Response by Phytocannabinoids

Similarly, extracts of the phytocannabinoids CBD and THC could attenuate the proliferation of activated lymphocytes and the secretion of pro-inflammatory IL-17, thereby increasing secretion of the anti-inflammatory IL-10 (86). Additionally, the endocannabinoid AEA and the phytocannabinoid THC could also induce immunosuppression in B cells as was examined in both primary and secondary *in vitro* plaque-forming cell assays of antibody formation (93). Many reports have shown that exogenously applied CBD suppresses transcription factors involved in inflammation like NFAT, AP-1, and NF-κB, which results in a broad repression of cytokines like IL-6, IL-1β, IL-1α, GM-CSF, and TNFα in diverse cells and tissues (140). These cytokines have a central role in the development of CRS in COVID-19. IL-6 promotes the differentiation of Th17 cells, which was shown to be suppressed by CBD (141). Moreover, CBD was shown to inhibit IFNγ (142).

A plethora of pre-clinical studies show that cannabinoids of certain cannabis strains can have an impact on the inflammatory response in mouse models of lung or inflammatory diseases, thus halting their progression. In a murine model of LPS-induced acute lung injury, CBD suppressed the vigorous immune response by three mechanisms: (i) inhibition of infiltration of leukocytes and neutrophils into lung tissue, (ii) inhibition of secretion of pro-inflammatory cytokines TNFα, IL-6, and the chemokines MCP-1 and MIP-2 into the BALF, (iii) inhibition of the activity of myeloperoxidase, an enzyme with antimicrobial activity abundantly expressed in neutrophils (143). In murine models of chronic asthma, cytokine levels of IL-4, IL-5, IL-6, IL-13, and TNFα were decreased by CBD, probably exerting its effect via the CB1 receptor. This led to the reduction of airway inflammation and fibrosis (144, 145). Moreover, the production of regulatory T cells were increased in murine models of inflammatory diseases (146).

**Figure 4:**
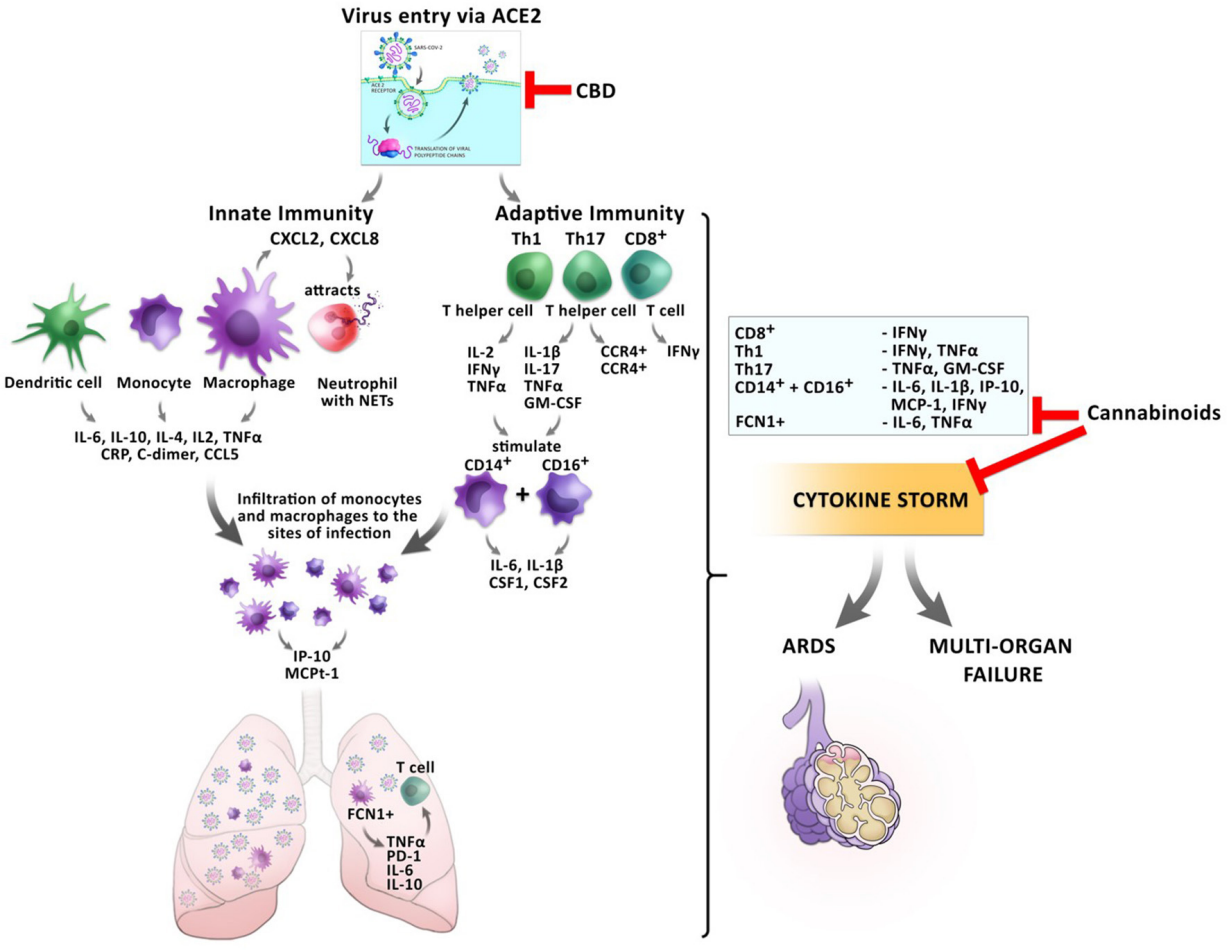
Impact of cannabinoids on inflammatory responses during a SARS-CoV2 infection. The entry of the virus via ACE2 can be inhibited by CBD, reducing the virus load inside the cells. Infection with the virus triggers a cascade of inflammatory responses of the innate and adaptive immunity. Monocytes and macrophages secrete cytokines and chemokines. Activated macrophages secrete CXCL2 and CXCL8 which attract neutrophils, which release NETs to the site of infection. Infiltrating FCN1+ macrophages secrete IL-6, IL-10, and TNFα in the lung, which leads to T cell apoptosis. CD8^+^ T cells secrete IFNγ and TNFα. T helper cells Th1 and Th17 stimulate CD14+ and CD16+ monocytes to secrete IL-6, IL-1β, and CSF1 and CSF2. This leads to the development of the cytokine storm, which might culminate in ARDS or multi-organ failure. Cannabinoids have the potential to inhibit the secretion of several pro-inflammatory cytokines resulting in prevention of CRS.

These anti-inflammatory actions of cannabis might be beneficial for the prevention of CRS before the host inflammatory response turns pathological during the transition from mild to critical disease in COVID-19 patients (Figure 4).

## Conclusion and Future Perspectives

According to the current state of available clinical data, most severe COVID-19 symptoms are related to CRS, which is also assumed to be responsible for the fatal outcome in COVID-19 patients. Here, we discuss the hypothesis that cannabinoids may have a great potential for the inhibition of hyperinflammation leading to CRS in COVID-19 patients. However, extensive evidence from pre-clinical and clinical trials are still missing but urgently needed. This is because in spite of the medicinal potential of cannabis, it may be used in harmful or abusive manner. Cannabis is the most widely used illicit drug in the world. The United Nations Office On Drugs and Crime World Drug Report (UNODC) from 2020 measured around 192 million users in 2018 (147). An increased use among older adults was seen in the US between after legalization 2015 and 2018 (148) and known cannabis users increased their usage during first lockdown in the Netherlands (149) and in the US (150). The most common route of cannabis administration is smoking with or without tobacco. This raises concern in relation to the development of a severe/critical disease state in COVID-19 patients because smoking tobacco upregulates ACE-2 which increases the entry rate of the virus into the cells and leads to a worse outcome (151). While in Europe still 77.2–90.9% prefer tobacco-based smoking (152), the use of alternative routes of cannabis administration like vaporizing or edibles have increased in the US since legalization (153). However, whether vaping has an advantage over smoking for the likelihood of an infection with SARS-CoV2 and its outcome are still unknown (154).

Moreover, severe cardiovascular events were reported after acute usage of herbal cannabis (155) including an elevated risk of myocardial infarction in the presence of *Angina pectoris* (156) and reported cardiovascular deaths in 26% of users between 2006 and 2010 (157). In adolescent users, regular herbal cannabis use can lead to irreversible cognitive decline including loss of short-term memory, mood disorders, and schizophrenia (158).

However, increasing evidence shows a positive impact of cannabidiol on chronic pain in adult patients, as an antiemetic in chemotherapy-induced nausea and vomiting and in improving spasticity in multiple sclerosis based on patient's reports as well as in sleep improvement and fibromyalgia (159). However, many more precisely targeted clinical studies need to be performed in order to evaluate the benefit/risk ratio for cannabinoids. All together, these concerns emphasize the need of deeper science-based data that will allow the appropriate use of cannabis for medicinal purposes. Our studies at the Medical Cannabis Research and Innovation Center follow this route. We aim to become more knowledgeable about the exact anti-inflammatory capability of the cannabinoid's components of a chosen strain with the lowest potential to drug abuse and the least adverse effects so that we can administer cannabinoids more accurately targeted to the patients.

## Author Contributions

IL-H and NP conceptualized the manuscript and established the writing consortium. AP wrote the section about the cannabinoid receptors. MA wrote the section about the anti-inflammatory properties of cannabinoids. HH, TP, and ES contributed to the writing and reviewing process. All authors contributed to the article and approved the submitted version.

## Conflict of Interest

The authors declare that the research was conducted in the absence of any commercial or financial relationships that could be construed as a potential conflict of interest.
